# Scleral Fixation of Posteriorly Dislocated Intraocular Lenses by 23-Gauge Vitrectomy without Anterior Segment Approach

**DOI:** 10.1155/2015/391619

**Published:** 2015-07-29

**Authors:** Jeroni Nadal, Bachar Kudsieh, Ricardo P. Casaroli-Marano

**Affiliations:** ^1^Institut Universitari Barraquer, Universidad Autònoma de Barcelona, Muntaner Street, No. 314, 08021 Barcelona, Spain; ^2^Department of Surgery, School of Medicine, University of Barcelona, Barcelona, Spain

## Abstract

*Background*. To evaluate visual outcomes, corneal changes, intraocular lens (IOL) stability, and complications after repositioning posteriorly dislocated IOLs and sulcus fixation with polyester sutures. *Design*. Prospective consecutive case series. *Setting*. Institut Universitari Barraquer. *Participants*. 25 eyes of 25 patients with posteriorly dislocated IOL. *Methods*. The patients underwent 23-gauge vitrectomy via the sulcus to rescue dislocated IOLs and fix them to the scleral wall with a previously looped nonabsorbable polyester suture. *Main Outcome Measures*. Best corrected visual acuity (BCVA) LogMAR, corneal astigmatism, endothelial cell count, IOL stability, and postoperative complications. *Results*. Mean follow-up time was 18.8 ± 10.9 months. Mean surgery time was 33 ± 2 minutes. Mean BCVA improved from 0.30 ± 0.48 before surgery to 0.18 ± 0.60 (*p* = 0.015) at 1 month, which persisted to 12 months (0.18 ± 0.60). Neither corneal astigmatism nor endothelial cell count showed alterations 1 year after surgery. Complications included IOL subluxation in 1 eye (4%), vitreous hemorrhage in 2 eyes (8%), transient hypotony in 2 eyes (8%), and cystic macular edema in 1 eye (4%). No patients presented retinal detachment. *Conclusion*. This surgical technique proved successful in the management of dislocated IOL. Functional results were good and the complications were easily resolved.

## 1. Introduction

Intraocular lens (IOL) dislocation into the vitreous cavity is a serious complication of cataract surgery, with a reported incidence of 0.1–1.7% [1]. Current management includes repositioning the lens in the sulcus when capsular support is sufficient or IOL removal when insufficient, in which case the IOL is removed through corneal limbal or corneal incisions or by enlarging one of the sclerotomies. However, these methods involve risks including producing up to 1 diopter (D) of corneal astigmatism [2], 8% of iris damage and hyphema [3], and 5% of retinal detachment [4].

Usually, aphakia is corrected secondarily by implantation of an anterior chamber intraocular lens (ACIOL) which entails the risk of 9.78% corneal endothelial compromise 3 years after iris fixation, 5.6% of iris pigment precipitates on the iris fixation [5], and 29% of cystoid macular edema (CME) after the implant of open loop [6]. Aphakia is also corrected by sutured posterior chamber lens (PCIOL), which entails the risk of 23% CME and 7% retinal detachment [6].

Recently a sutureless intrascleral PCIOL fixation technique is done to correct aphakia [7, 8]; this technique entails the risk of subsequent IOL dislocation of 3.6% and 1.5% of iris capture of IOL during mean follow-up time of 7 months [8]; this percentage can rise to 12.5% of IOL dislocation during a mean follow-up of 1 year [7].

The aims of this study were first to describe a more conservative and rational surgical technique to manage posteriorly dislocated IOL by repositioning and fixation to the scleral wall using nonabsorbable polyester sutures and then to analyze visual outcomes and postoperative complications, in order to assess whether this technique avoids the most commonly observed complications associated with other methods.

## 2. Patients and Methods

We performed a prospective study of a consecutive case series comprising 25 eyes of 25 patients who underwent 23-gauge vitrectomy via the sulcus to manage posteriorly dislocated IOLs. All 25 eye surgeries were performed by the same surgeon (JN) between 2011 and 2014 in the Barraquer Eye Center, Barcelona, Spain. Institutional Ethics Committee for Clinical Research approval was obtained for this study. It was conducted following the postulates of the Declaration of Helsinki and written informed consent was obtained from each patient after receiving full information about the procedures involved.

The criterion for inclusion was dislocation of posterior chamber two-haptic IOL into the vitreous cavity. Exclusion criteria were the existence of sufficient anterior capsular support to allow repositioning the IOL in the ciliary sulcus and follow-up period less than 12 months after surgery.

All patients underwent a monocular best corrected visual acuity (BCVA) examination, measured by LogMAR chart, before and after refraction test, and Amsler Chart test to identify metamorphopsia and macular optical coherence tomography (OCT) (Carl Zeiss Meditec, Dublin, CA) was done in case of the presence of metamorphopsia or reduced BCVA during the follow-up. They also underwent slit lamp eye examination, funduscopy under pharmacologic mydriasis, intraocular pressure measurement (Goldmann), B-mode ultrasound echography, corneal topography (OCT CASIA SS-1000, Tomey, Nagoya, Japan), and endothelial cell count by specular microscopy (Konan SP 9000, Konan, Inc., Nishinomiya, Japan).

The variables collected before surgery included age, gender, lens type, possible cause of dislocation, BCVA, corneal astigmatism steep axis power (*K*_max_) and flat axis power (*K*_min_), and endothelial cell count (cells/mm^2^).

### 2.1. Surgical Technique

**Figure 1:**
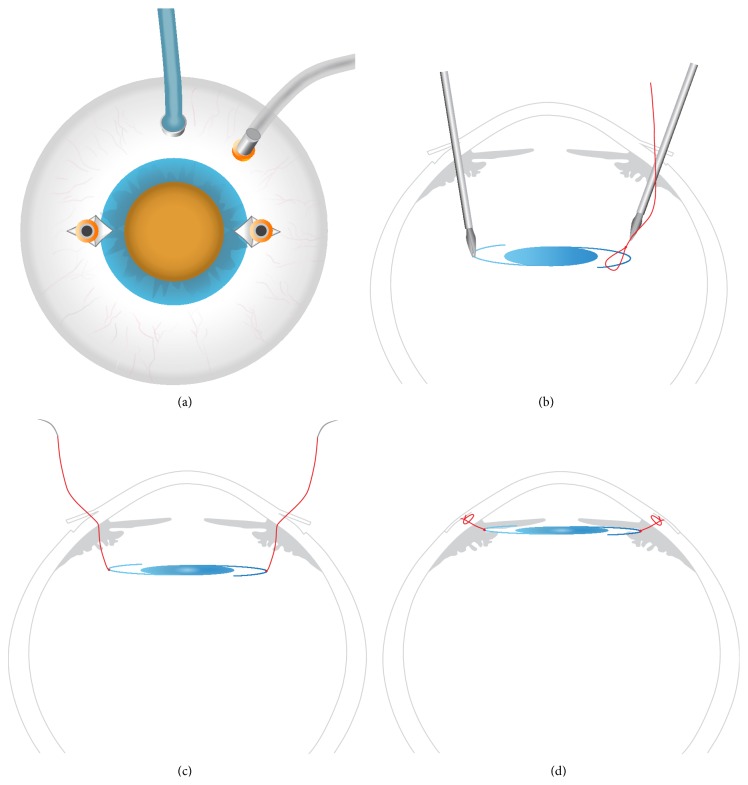
(a) Two scleral limbal-based flaps at 3 and 9 o'clock were created. Three 23-gauge vitrectomy ports were introduced, two passing through the scleral flaps, with an accessory 25-gauge light source introduced at 12 o'clock. (b) After complete vitrectomy, the lens was raised to the middle of the vitreous cavity using two peeling forceps. Each haptic was snared by a previously prepared loop; then the suture was tensed around the middle of the haptic. (c) Once the two haptics of the lens were captured, the lens was repositioned in the sulcus by simply tensing the two sutures. (d) Once the two haptics of the lens were repositioned in the sulcus, the sclerotomies were closed with the same sutures as those holding the lens.

**Figure 2:**
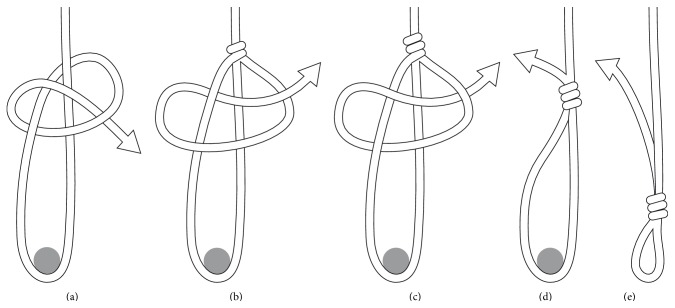
Five-steep diagram showing in a simple way how the adjustable loop is done.

Peritomy and wet cautery of the sclera at 3 and 9 o'clock were performed. Two triangular partial thickness limbal-based scleral flaps measuring 2 mm by 2.5 mm were created at 1.5 mm from the limbus at 3 and 9 o'clock. Three 23-gauge ports were introduced, two of the three ports passing thorough the scleral wall under the scleral flaps. The infusion port was introduced transconjunctivally at 2 o'clock. Then, 23-gauge vitrectomy was performed to remove the vitreous gel and free the lens from possible vitreous adhesions and the rest of the capsular bag. After complete vitrectomy, a halogen light source was introduced (Figure 1(a)) and the two ports previously positioned under the scleral flaps were removed, and the sclerotomies were enlarged using a 23-gauge sclerotome. Two 23-gauge peeling forceps were introduced into the vitreous cavity and used to gently raise the lens to the middle of the vitreous cavity so the lens and the haptics could be clearly visualized. Holding the lens with one of the two forceps in the middle of the vitreous cavity, a previously prepared adjustable looped (Figure 2) nonabsorbable polyester 10-0 suture (MERSILENE, Ethicon, Scotland, UK) (please see the supplementary materials video demonstrating the loop preparation details and the surgery in Supplementary Material available online at http://dx.doi.org/10.1155/2015/391619) was introduced with the other forceps and the lens haptic was snared in the loop (Figure 1(b)). The suture was then tensed around one haptic and the same maneuver was then repeated to capture the other haptic (Figure 1(c)). Once the two haptics of the lens were captured, the lens was repositioned in the sulcus by simply tightening the two sutures. The two sclerotomies were closed with the same sutures as those holding the lens (Figure 1(d)). All patients were discharged 6 to 8 hours after surgery under treatment with dexamethasone 1 mg/mL and tobramycin 3 mg/mL eye drops (Tobradex, Alcon Cusi, Barcelona, Spain).

The duration of surgery was recorded beginning at the moment of extraocular suture preparation and finishing when the eye speculum was removed. All patients underwent postoperative follow-up at day 1, 1 week, 1 month, 6 months, and 12 months and yearly thereafter. The following variables were recorded after surgery: 1-month BCVA, 12-month BCVA, 12-month astigmatism *K*_max_ and *K*_min_, 12-month endothelial cell count, and lens stability by slit lamp examination after pharmacological mydriasis. Postoperative complications such as vitreous hemorrhage, CME, and presence of hypotony during the first week, defined as IOP less than 5 mmHg, were also recorded.

### 2.2. Statistical Analysis

Statistical analysis was performed using SPSS 17.0 (SPSS Inc., Chicago, Illinois, USA). Kolmogorov-Smirnov test was used to test the normal distribution of continuous variables. Paired* t*-test was used to compare BCVA, endothelial cell count, and astigmatism before and after surgery. Differences with a *p* value < 0.05 were considered statistically significant.

## 3. Results

In total, 25 eye surgeries were included in this analysis. Mean age was 61.2 ± 21.6 years. Mean follow-up was 18.8 ± 10.9 months, range 18–48 months. Baseline patient characteristics are shown in Table 1.

The adjustable suture preparation technique was practiced in the wet lab before performing the surgery in human eyes in order to shorten the surgery time and fasten the learning curve. Mean surgery time was 33 ± 2 minutes, range 30–37 minutes.

Mean BCVA improved from 0.30 ± 0.48 before surgery to 0.18 ± 0.60 (*p* = 0.015, paired* t*-test) at 1 month. No statistically significant difference was found between BCVA at one month and BCVA at 12 months (0.18 ± 0.60) (*p* = 0.486). No statistically significant difference was found between *K*_max_ before surgery (44.0 ± 2.2) and at 12 months (44.2 ± 1.9) (*p* = 0.613). Similarly, we found no statistically significant difference between *K*_min_ before surgery (42.1 ± 1.8) and at 12 months (42.3 ± 1.6) (*p* = 0.361). Finally, no statistically significant difference was found between endothelial cell count before surgery (1,858 ± 698) and that at 12 months (1,835 ± 696) (*p* = 0.685).

**Figure 3:**
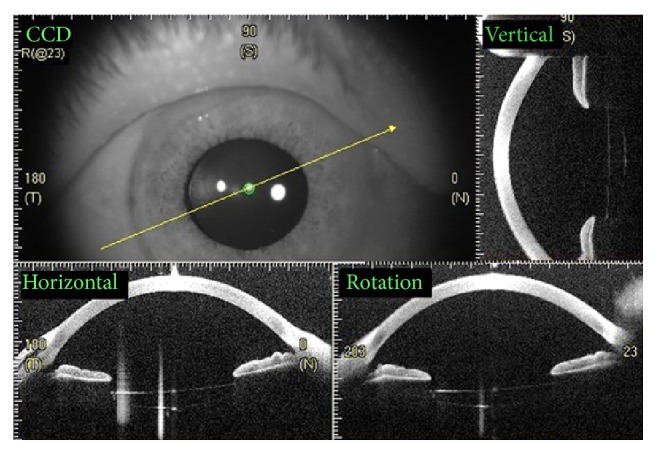
Anterior segment OCT showing IOL stability and centration at 1 year after surgery. Vertical, horizontal, and rotational sections are shown.

Clinical lens stability and centration was observed by slit lamp and anterior segment OCT examination in 24 (96%) patients (Figure 3); one of the 25 patients presented a decentered lens during the first month after surgery due to a loose suture and needed a simple additional intervention to reopen the scleral flap, tense the suture, and recenter the lens. This patient did not present new subluxation or any other complication during the follow-up period. Vitreous hemorrhage was seen in 2 (8%) eyes during the first month after surgery. Vitrectomy was performed in one patient to clear the hemorrhage and spontaneous resolution was observed in the other; both had good visual outcomes, with 12-month BCVA of 0.18 and 0.10, respectively.

Two patients (8%) presented hypotony within the first 3 days of surgery but did not present sclerotomy site leakage. The hypotony resolved spontaneously at day 5 after surgery without additional treatment in both cases. CME was observed in one patient (4%) during the first month after surgery; complete resolution of the edema was obtained with subtenon triamcinolone acetonide (40 mg/mL) injection, but the patient presented reduced BCVA (0.40) at the end of clinical follow-up. None of the patients in this cohort presented retinal detachment.

## 4. Discussion

The present study describes visual outcomes and complications of a surgical technique that could avoid the most commonly observed complications associated with other methods, especially corneal complications associated with ACIOL implantation or sutured PCIOL. In addition, this technique reflects a more conservative approach that allows faster postoperative visual recovery.

Maguire et al. [9] described a surgical technique to manage dislocated IOL in six patients in which preprepared loops of 10-0 Prolene sutures and sclerotomy incisions for vitrectomy were utilized for fixation of posteriorly dislocated IOL. Our surgical approach introduced important modifications and improvements in other aspects. The use of microsurgical instruments helps to reduce wound leakage and inflammation. In addition, an accessory light facilitates bimanual maneuvers and lens handling during surgery. Furthermore, the creation of scleral flaps is important to prevent suture erosion and exposure to ultraviolet light that could damage the suture and cause subsequent IOL dislocation. Lastly, we used nonabsorbable polyester sutures, rather than the Prolene used by Maguire et al., which could be more resistant and last longer [10].

Mean surgery time was 33 ± 2 minutes; we believe that this technique is simple to realize and has a short learning curve due to two main factors: the first resides in the possibility of practicing the adjustable loop preparation in a wet lab and the second factor is that the lens handling in the vitreous cavity is relatively easy if done by retina surgeon familiar with bimanual maneuvers.

In our series, mean BCVA improved by almost 3 lines. This improvement was achieved during the first month after surgery and maintained without changes during the first year after surgery, indicating rapid and persisted visual recovery. Our results compare favorably with the improved BCVA of 0.18 (LogMAR) reported by Mittra et al. [11] when vitrectomy, IOL* pars plana* removal, and IOL exchange to ACIOL were performed. Also our results are similar to the final visual acuities of 0.40 (LogMAR) reported by Sarrafizadeh et al. [6] and the visual acuity of 0.48 (LogMAR) reported by Vote et al. [12] using* pars plana* vitrectomy and IOL exchange to sutured PCIOL placement.

Interestingly, our approach did not induce further corneal astigmatism. In our cohort, topography did not show any changes after surgery, as the technique does not involve corneal incisions to insert the IOL, which can produce astigmatism of 1 D as reported by Kwon et al. [2] after corneal, limbal, or scleral incisions were done to insert an ACIOL. Also corneal astigmatism was produced after clear cornea incisions done to PCIOL scleral fixation as reported by Hoffman et al. [13]. In addition, no changes were observed in endothelial cell count, another great advantage to take into account compared with the usual endothelial cell loss associated with ACIOL implantation, as reported by Chen et al. [5]. Other previous studies have reported mean endothelial cell loss of 9.78% three years after iris claw ACIOL implantation [5] or 2% after open loop ACIOL implantation [14].

IOL stability and centration was observed in 24 eyes (96%) (Figure 2) and subluxation was seen in one eye (4%) at one month after surgery. In this case the lens was an acrylic two-haptic lens (AcrySof MA60BM, Alcon, Texas, Fort Worth, USA), and we believe that this complication was partly attributable to the flexible nature of its haptics. IOL decentration has been observed in 4.8%–6% of cases receiving iris claw and open loop ACIOLs, respectively [14]; also the dislocation was observed in 12.5% of cases of sutureless scleral fixation technique described by Wilgucki et al. [7] during one-year follow-up and up to 3.6% as referred by Scharioth et al. [8] during a follow-up time of 7 months. Another advantage to mention is that our described surgical technique was performed in two-haptic IOL, whether one piece or three pieces, while the intrascleral fixation technique described by Scharioth et al. [8] and Wilgucki et al. [7] was done only in three-piece IOL.

No suture breakage occurred in our cohort during the mean follow-up time of 18.8 ± 10.9 months and ranging in some cases till 48 months. Vote et al. [12] reported suture breakage and subsequent new IOL dislocation as the most common complication, found in 17 eyes (27.9%) of their series undergoing* pars plana* vitrectomy and scleral fixed sutured PCIOL. We consider that a longer follow-up time is needed to prove that nonabsorbable polyester sutures are less susceptible to breakage and provide long-term lens stability, which is critical to ongoing use of this technique.

Our surgical technique presented few postoperative complications which were treated with conservative measures. Vitreous hemorrhage was seen in 2 (8%) eyes, probably due to intraoperative manipulation of the ciliary body. Steinmetz et al. [15] reported this complication in 3 (5%) eyes due to possible iris or ciliary body manipulation when the IOL was removed by* pars plana* vitrectomy. The incidence of retinal detachment after vitrectomy for dislocated IOL varies from 0% to 6.3% [16, 17]; minimal manipulation of the vitreous base and 23-gauge incision used to perform our technique may minimize the risk of retinal tear or detachment, neither of which occurred in our patients. CME has been reported in 22% of eyes undergoing* pars plana* vitrectomy, with IOL removal and exchange to open loop ACIOL [14]. In our cohort, minimal surgical incisions and less contact of the IOL with the uvea could explain the reduced number of eyes suffering CME. Finally, hypotony was observed in 2 (8%) eyes without scleral or conjunctival flap leakage. However, complete spontaneous resolution was observed at day 5 after surgery; a temporary slight suppression of the ciliary body may be considered the cause of this complication.

In conclusion, we consider the surgical technique described above a simple and an easy learning technique. The good visual outcomes, the easily treatable complications, and the use of the same IOL made the surgical technique our first choice to be performed by all the surgeons in our retina department. It is important to acknowledge that the major limitation of our study is the relatively short mean follow-up of 18.8 ± 10.9 months which makes it unable to answer the question whether suture breakage and the subsequent lens stability may limit the long-term use of this technique.

## Supplementary Material

Helped by the arm of the eye speculum, the adjustable node is done in four steps maneuver.
Surgery starts with the peritomy and cautery of the sclera at 3 and 9 o'clock. Two triangular partial thickness limbal-based scleral are created. Three ports are introduced, two of the three ports passing under the scleral flaps. The infusion port was introduced transconjunctivally and after complete vitrectomy, a halogen light source is introduced and the two ports previously positioned under the scleral flaps are removed. Two forceps are introduced into the vitreous cavity and used to gently raise the lens to the middle of the vitreous cavity so the lens and the haptics can visualize. Holding the lens with one of the two forceps in the middle of the vitreous cavity, the adjustable suture is introduced with the other forceps and the lens haptic was snared in the loop that is tensed around one haptic. Once the two haptics of the lens were captured, the lens was repositioned in the sulcus by tightening the two sutures. The two sclerotomies are closed with the same sutures.

## Figures and Tables

**Table 1:** Baseline characteristics of patients undergoing 23-gauge vitrectomy via the sulcus to manage posteriorly dislocated IOL.

	Cases (*n* = 25)
Female sex: *n* (%)	8 (32%)

Age in years: mean ± SD	61.2 ± 21.6

Endothelial cell count (cells/mm^2^): mean ± SD	1,858 ± 698

BCVA (LogMAR chart): mean ± SD	0.30 ± 0.48

Astigmatism (D): mean ± SD	
*K*_max⁡_	(44.0 ± 2.2)
*K*_min⁡_	(42.1 ± 1.8)

Cause of dislocated IOL	
Trauma	3 (12%)
Pseudoexfoliation syndrome	3 (12%)
Marfan syndrome	1 (4%)
Intraoperative complications	1 (4%)
Unknown	17 (68%)

IOL type	
AcrySof SA60AT^†^	4 (16%)
AcrySof MA60BM^†^	5 (20%)
CZ70BD^†^	2 (8%)
C-flex 570C-IOL^‡^	1 (4%)
722Y^§^	1 (4%)
MORCHER 67 G^¶^	1 (4%)
Unknown	11 (44%)

SD: standard deviation; BCVA: best corrected visual acuity; D: diopters; *K*_max⁡_: steep axis; *K*_min⁡_: flat axis; †: Alcon, Texas, Fort Worth, USA; ‡: Rayner, Hove, UK; §: Advanced Medical Optics Inc., Santa Ana, California, USA; ¶: MORCHER Gmbh, Stuttgart, Germany.
